# Supplementary figures and images for: African green monkeys avoid SIV disease progression by preventing intestinal dysfunction and maintaining mucosal barrier integrity
Source: PLoS Pathog. 2020 Mar 2;16(3):e1008333. doi: 10.1371/journal.ppat.1008333 (PMC7077871; doi:10.1371/journal.ppat.1008333)

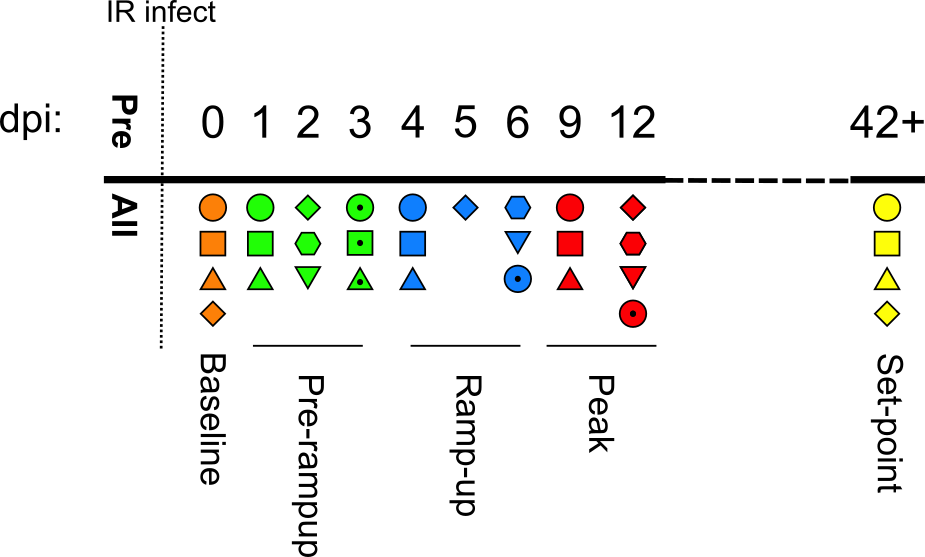

Supplement: S1 Fig — AGMs were serially euthanized throughout the acute and early chronic SIV infection and were divided into the following groups: (i) preinfection (baseline); (ii) preramp-up (1–3 dpi); (iii) ramp-up (4–6 dpi); (iv) peak (9–12 dpi); (v) set-point (46–55 dpi). Each group is assigned a corresponding color: orange (baseline), green (preramp), blue (ramp-up), red (peak) and yellow (set-point). (TIF) [file ppat.1008333.s006.tif]

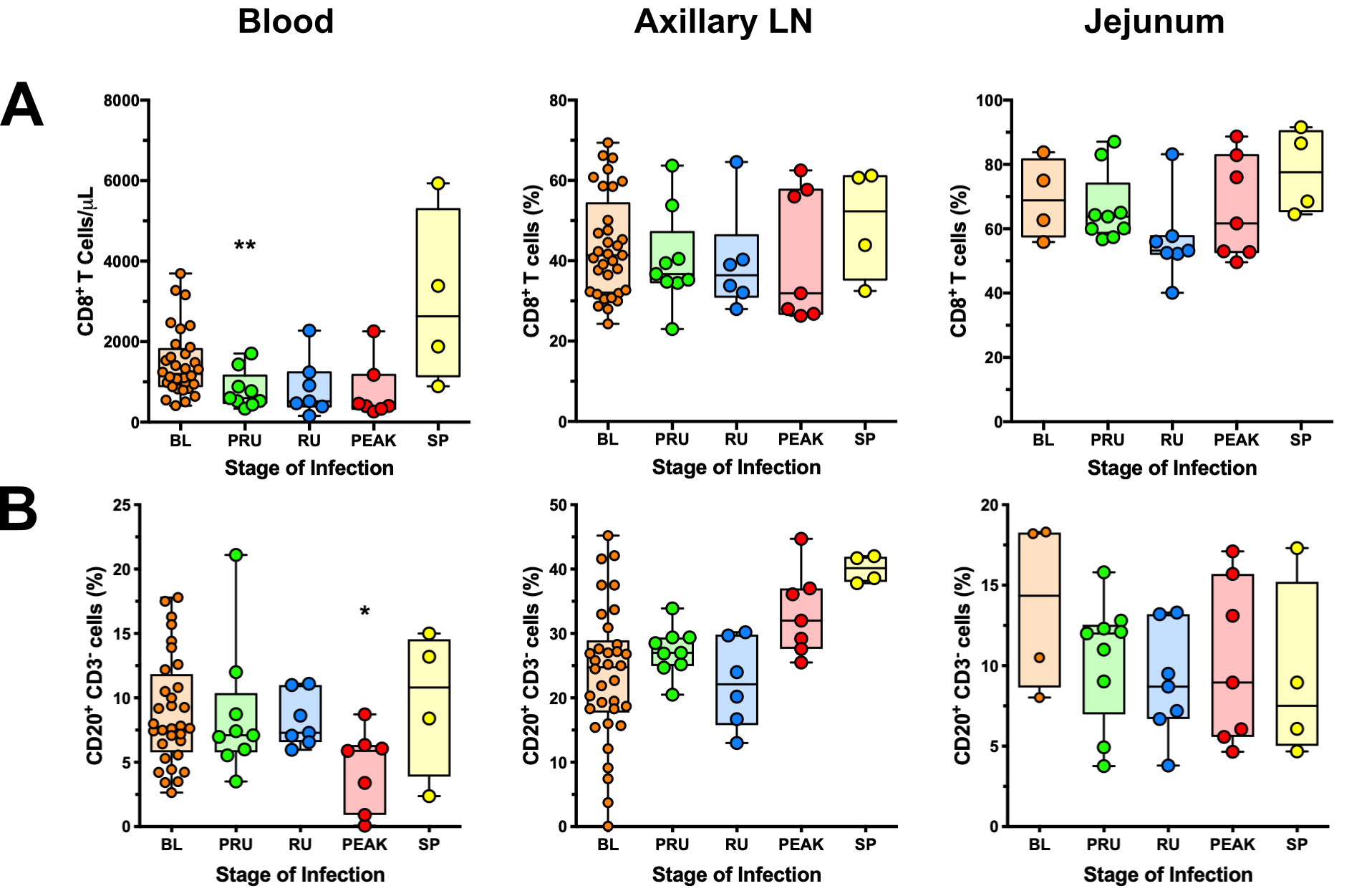

Supplement: S2 Fig — Total populations of (A) CD8+ T cells; and (B) CD20+ B cells isolated from blood, axillary LN and jejunum. The values for blood represent absolute counts, while the values in the jejunum and LN represent percent populations. The five groups are based on the days postinfection, with: BL (baseline, preinfection), PRU (preramp, 1–3 dpi) RU (ramp-up, 4–6 dpi), PEAK (peak, 9-12dpi) and SP (set-point, 46–55 dpi). Each group is assigned a corresponding color: orange (baseline), green (preramp), blue (ramp-up), red (peak) and yellow (set-point). Asterisks indicates statistical significance when compared to baseline values, with * = p<0.05; ** = p<0.01. (TIF) [file ppat.1008333.s007.tif]

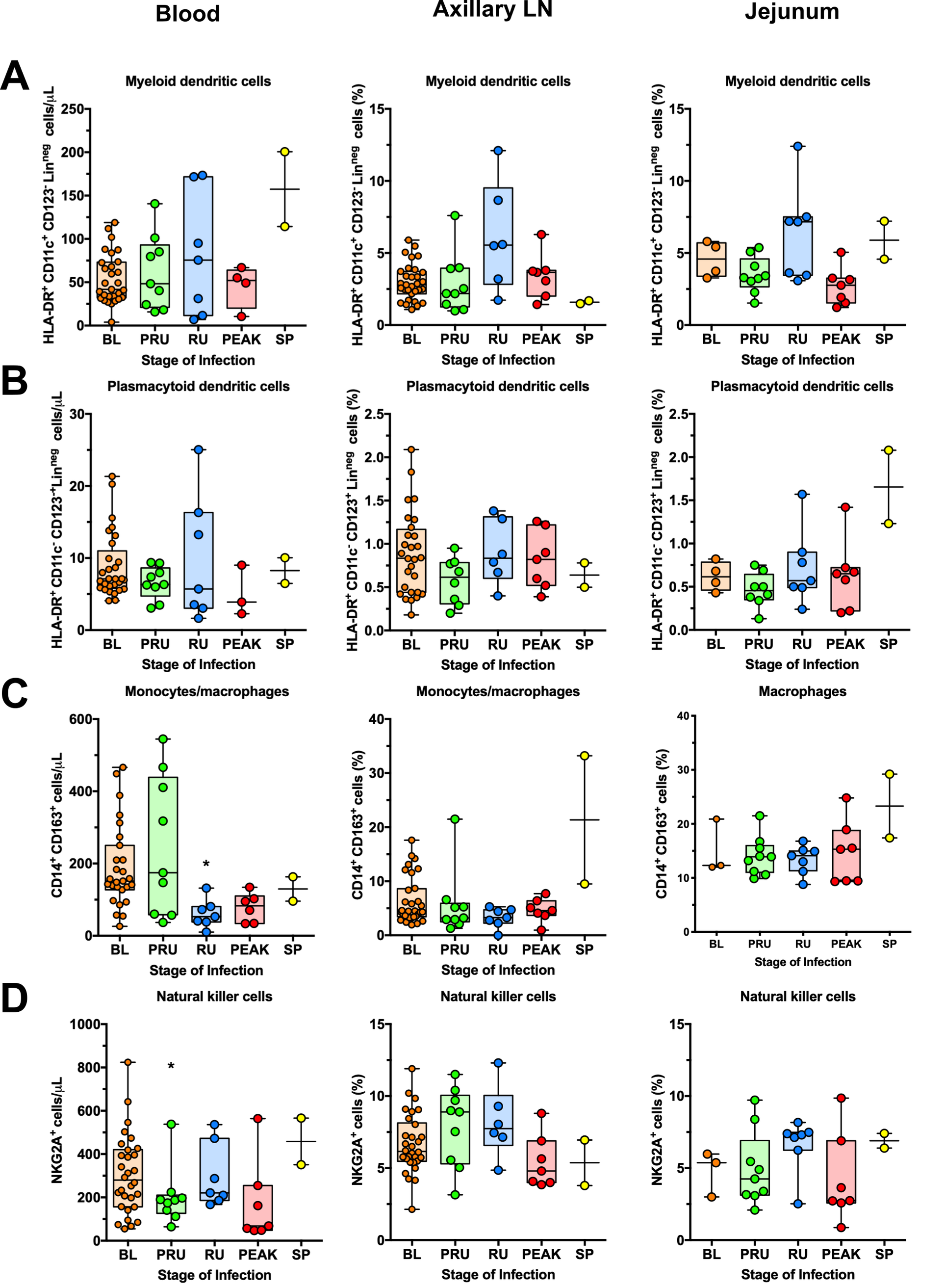

Supplement: S3 Fig — The flow cytometry analysis encompassed multiple immune cell subtypes, including: (A) myeloid dendritic cells; (B) plasmacytoid dendritic cells; (C) monocytes/macrophages (CD14+ CD163+); and (D) natural killer cells (NKG2A+). These cells were isolated from a variety of different tissues, including blood, jejunum and axillary LN. The five different time groups are based on the days postinfection, with: BL (baseline, preinfection), PRU (preramp, 1–3 dpi) RU (ramp-up, 4–6 dpi), PEAK (peak, 9-12dpi) and SP (set-point, 46–55 dpi). Each time group is assigned a corresponding color: orange (baseline), green (preramp), blue (ramp-up), red (peak) and yellow (set-point). Asterisks indicates statistical significance when compared to baseline values, with * = p<0.05. (TIF) [file ppat.1008333.s008.tif]

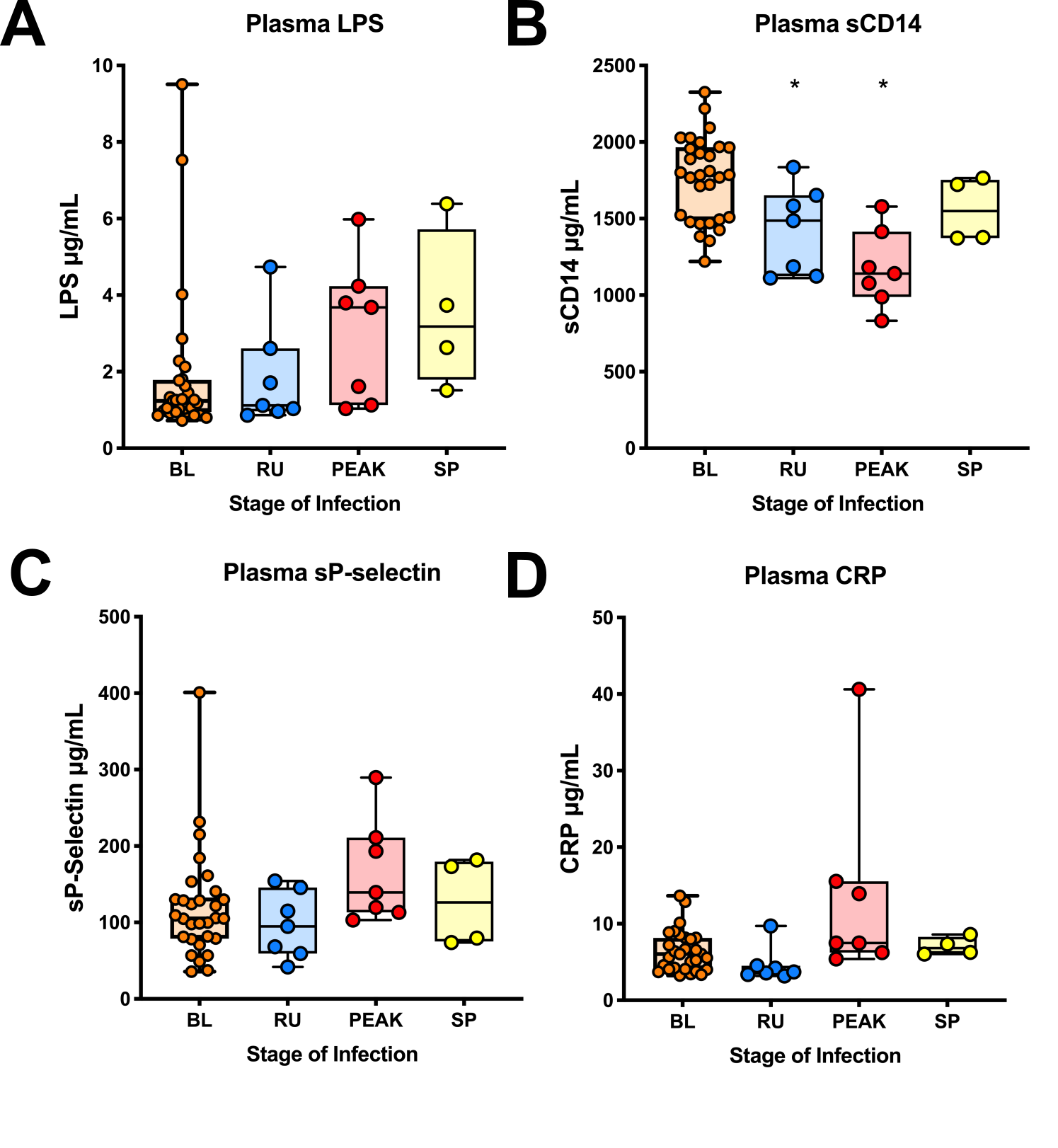

Supplement: S4 Fig — Plasma from each animal was tested using ELISA for: (A) lipopolysaccharide (LPS); (B) C-reactive protein (CRP); (C) soluble P-selectin (sP-selectin); and (D) soluble CD14 (sCD14). The values shown represent a total fold change in from baseline levels for each animal. The five groups are based on the days postinfection, with: BL (baseline, preinfection, orange), PRU (preramp, 1–3 dpi, green) RU (ramp-up, 4–6 dpi, blue), PEAK (peak, 9-12dpi, red) and SP (set-point, 46–55 dpi, yellow). Asterisks indicates statistical significance when compared to baseline values, with * = p<0.05; ** = p<0.01. (TIF) [file ppat.1008333.s009.tif]

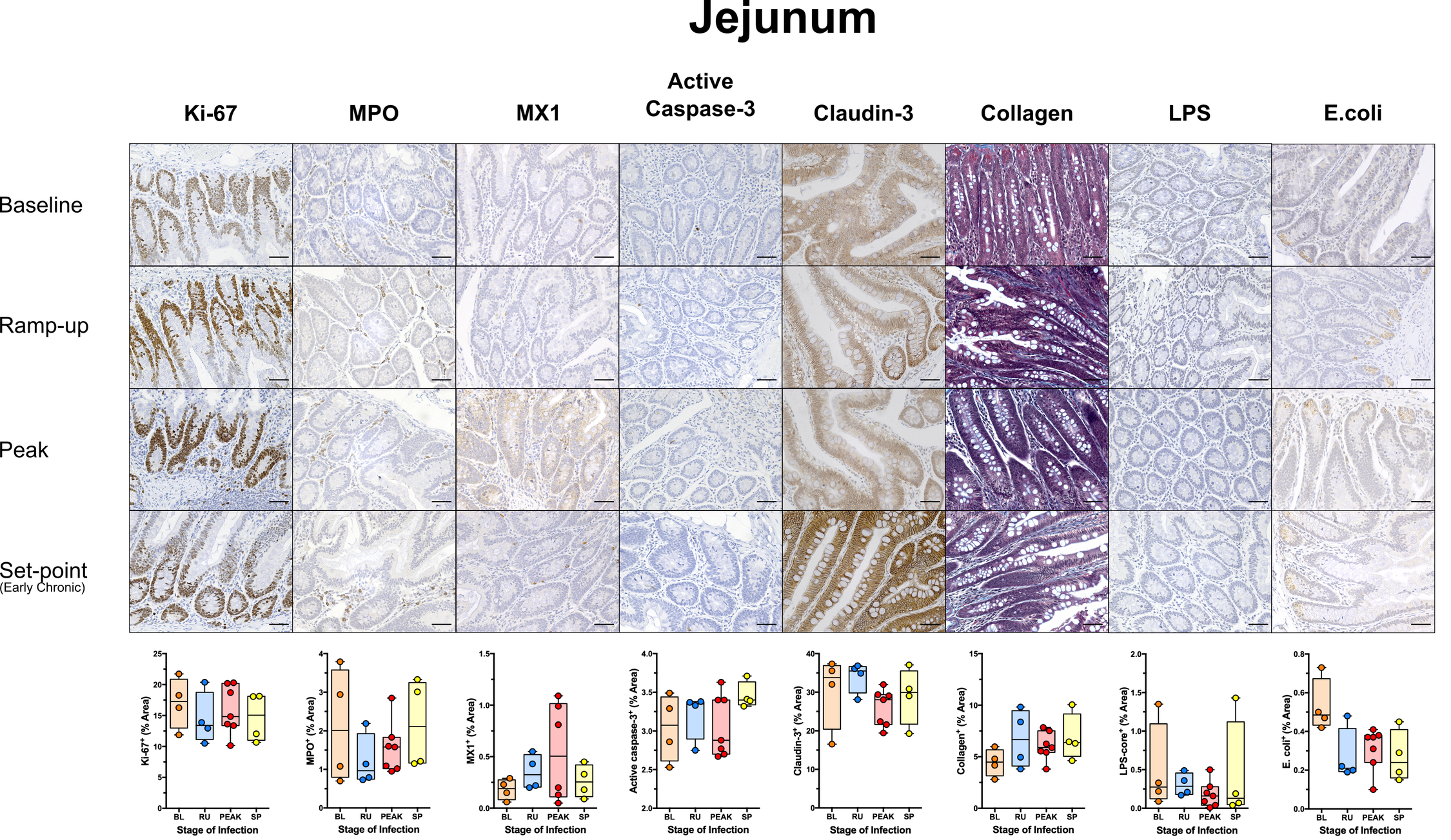

Supplement: S5 Fig — DAB-based IHC for the same array of markers that were used for the colon and axillary LN. In all the images, positive DAB signal is shown in brown, with the remaining tissue counterstained blue. Below are shown quantifications of positive signal within the image. The quantification for each animal represents the average of the values from 9–12 individual image quantifications. Villi enterocytes were included in all quantifications. The four different time groups are based on the days postinfection, with: BL (baseline, preinfection, orange), PRU (preramp, 1–3 dpi, green) RU (ramp-up, 4–6 dpi, blue), PEAK (peak, 9-12dpi, red) and SP (set-point, 46–55 dpi, yellow). All quantifications were performed using FIJI version—1.0. Asterisks indicate statistical significance p<0.05. All images were captured at 200X magnification using an AxioImager M1 bright-field microscope equipped with an AxioCam MRc5. Scale bar: 100 μm. (TIF) [file ppat.1008333.s010.tif]

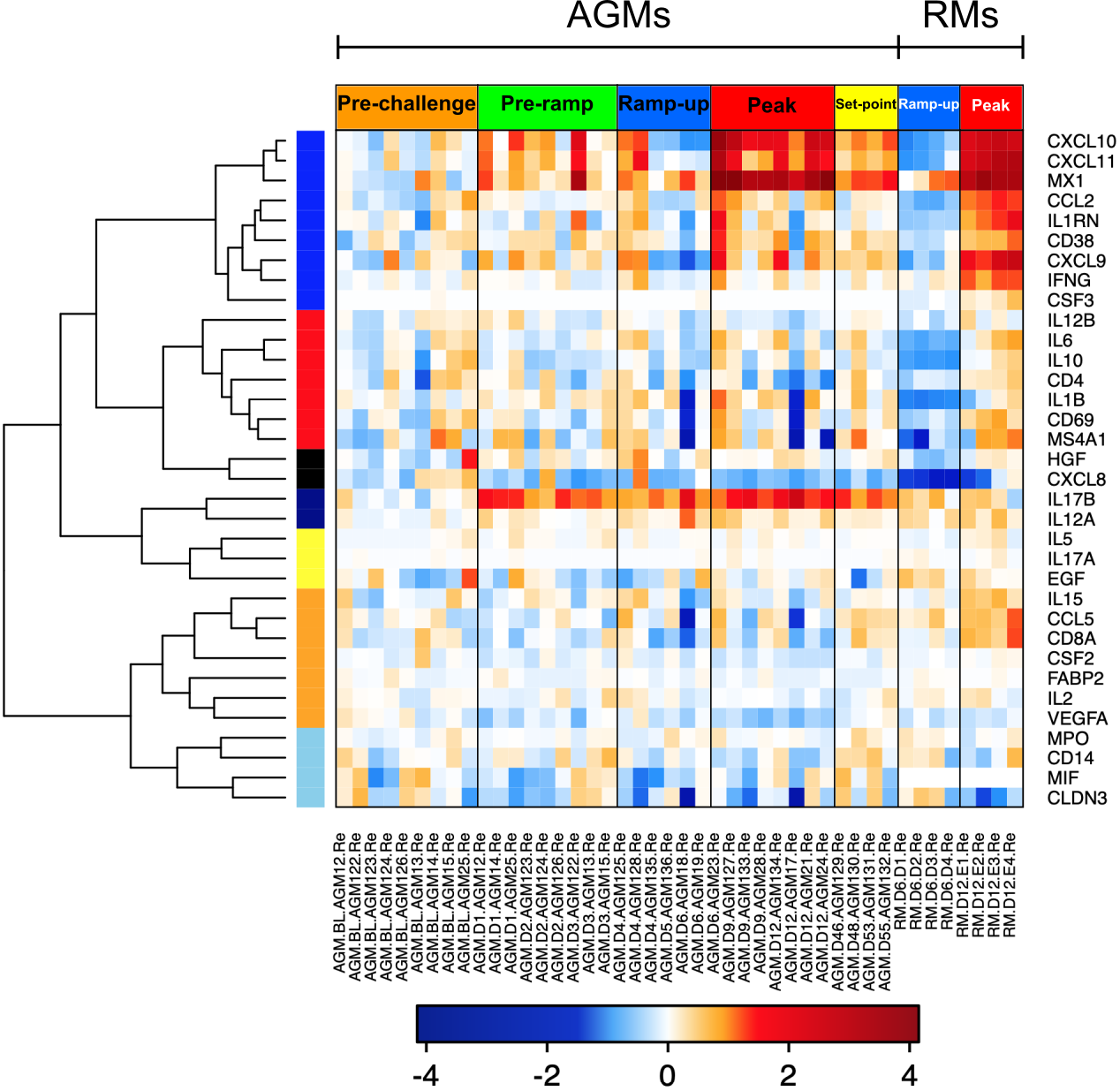

Supplement: S6 Fig — RNAseq data from AGM and RM gut tissue displayed as a heatmap showing gene expression changes in specific genes of interest associated with biological processes related to SIV-infection and the host immune response. The level of alteration of gene expression is shown in blue (downregulation) and red (upregulation), with genes clustered using a Spearman correlation, with the dendrogram showing relationship dispalyed on the left. Animal numbers are shown below the heatmap along with dpi, while the time groups are shown above the heatmap, with the colors indicating the groups: orange (, BL, baseline), green (PRU, preramp), blue (RU, ramp-up), red (peak), yellow (SP, set-point). The data for the time groups of the AGMs are listed in black text on the left, while the data from the equivalent RM time groups are listed in white text on the right. (TIF) [file ppat.1008333.s011.tif]

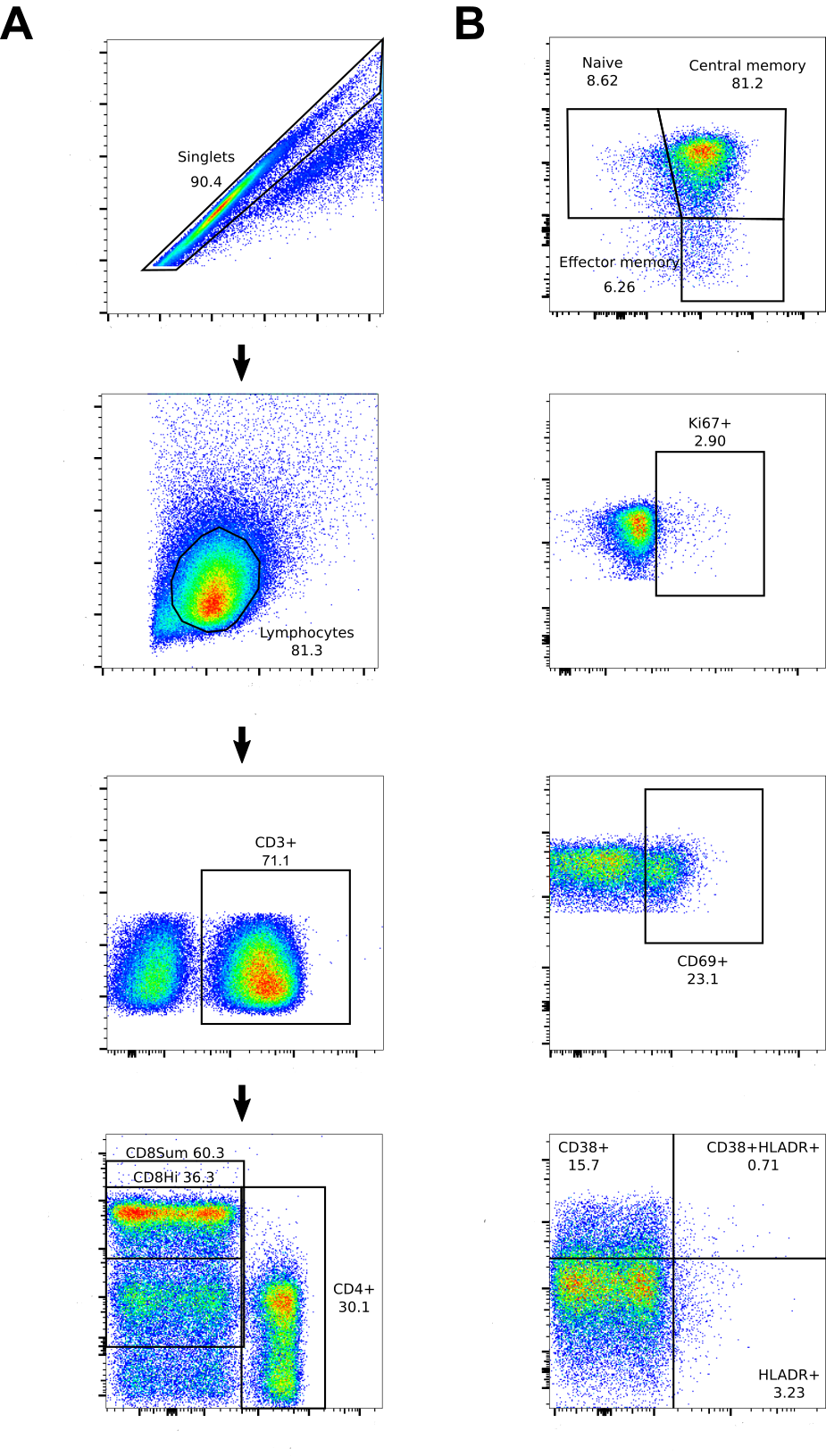

Supplement: S7 Fig — (A) Gating strategy used to delineate primary T-cell populations (CD3+, CD4+, CD8+) T-cell populations. (B) Gating strategies to delineate the secondary T cell populations (EM, CM and naïve), Ki-67+ T cells, CD69+ T cells, and HLA-DR+ CD38+ T cells. All plots shown in (B) are CD4+ T cells, but the same gating strategies were used for CD8+ T cells. All gates were generated using Flowjo software version 10.1r5 (Tree Star Inc, Ashland, OR). (TIF) [file ppat.1008333.s012.tif]

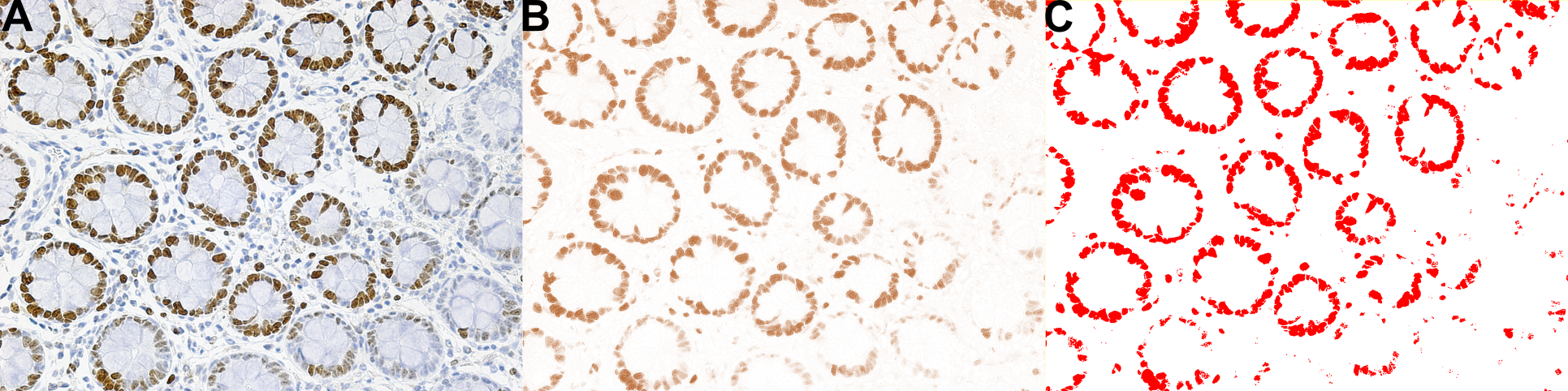

Supplement: S8 Fig — To quantify the DAB stain, each raw image (A) was processed using the Color Deconvolution 1.7 plugin for FIJI v.1.0. The preset DAB settings were selected, and the software separated the image into 3 color channels, with the brown channel representing the positive DAB signal (B). A threshold was then manually applied to the brown channel image to remove background coloration (C). Finally, the area of each image representing the DAB signal above threshold was measured as a percentage of the total area of the image. All images were captured at 200X magnification using an AxioImager M1 brightfield microscope equipped with an AxioCam MRc5. All image manipulations and measurements were done with FIJI v.1.0. (TIF) [file ppat.1008333.s013.tif]

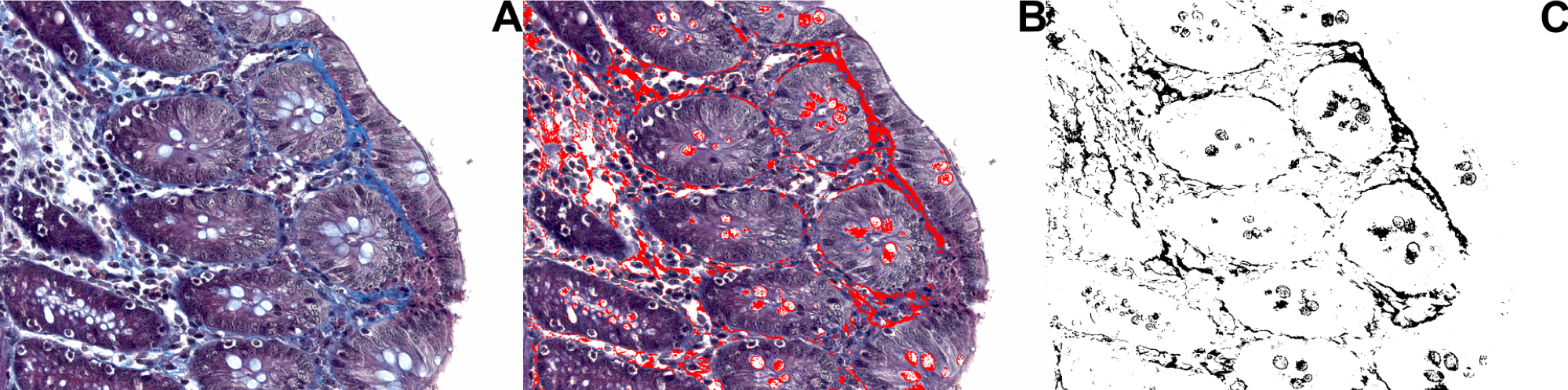

Supplement: S9 Fig — To quantify the amount of collagen in each tissue, each raw image (A) was processed with the built-in Color Threshold function in FIJI v.1.0. Using this feature, first the collagen was isolated from the rest of the image by adjusting the Hue value of the Color Threshold function to only encompass the blue of the collagen (B). It should be noted that the blue dye was also partially taken up by the goblet cells in the mucosal epithelium. After setting the Color Threshold, all background colors were removed, and the image was transformed into black and white (C). This eliminates all area of the image that is not blue coloration, which then can be measured by setting an intensity threshold to select all black area in the image. All images were captured at 200X magnification using an AxioImager M1 brightfield microscope equipped with an AxioCam MRc5. All image manipulations and measurements were done with FIJI v.1.0. (TIF) [file ppat.1008333.s014.tif]

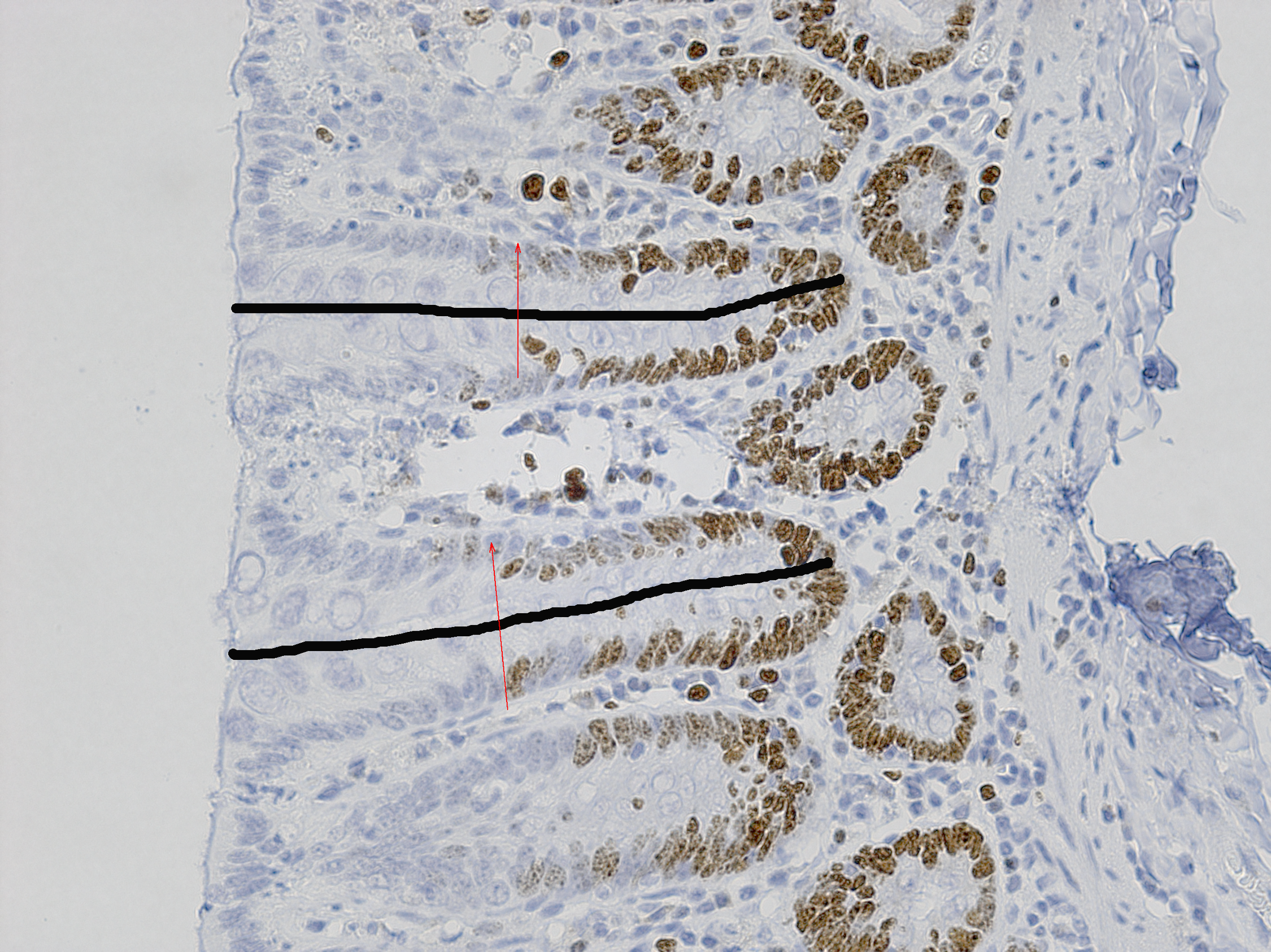

Supplement: S10 Fig — To generate an approximation of the levels of on-going proliferation in the gut epithelium, we captured images of individual crypts in tissue sections stained with Ki-67. As epithelial proliferation is localized to the base of the crypts, we measured both the total length of the crypt (total black line) and the length of the crypt with Ki-67+ epithelial cells (black line below the bisecting green arrow). Since increased levels of proliferation should result in an increase in the total number of Ki-67+ cells along the crypt, taking the ratio of the areas of the line allowed us to normalize proliferation across multiple crypts. All individual images were captured at 200X magnification using an AxioImager M1 brightfield microscope equipped with an AxioCam MRc5. All image manipulations and measurements were done with FIJI v.1.0. (TIF) [file ppat.1008333.s015.tif]

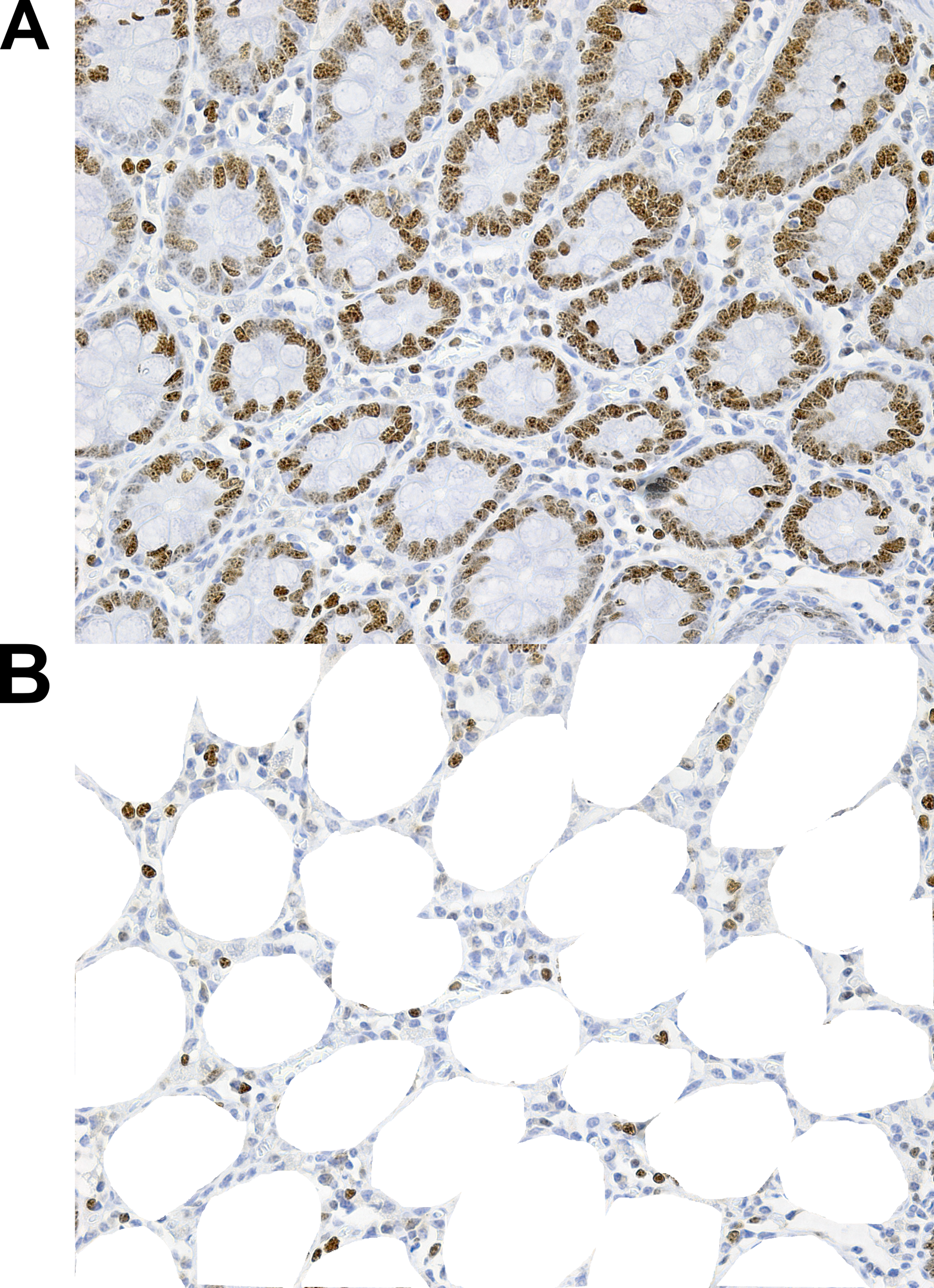

Supplement: S11 Fig — To measure the Ki-67 expression by cells in the lamina propria, all the epithelial cells were manually removed from the images. By overlaying white coloration on the epithelial sections of the crypts, they were excluded from the thresholding for positive DAB signal (S8 Fig). Then, a threshold could be applied to the area within the lamina propria alone and the total DAB signal measured as normal. All individual images were captured at 200X magnification using an AxioImager M1 brightfield microscope equipped with an AxioCam MRc5. All image manipulations and measurements were done with FIJI v.1.0. (TIF) [file ppat.1008333.s016.tif]

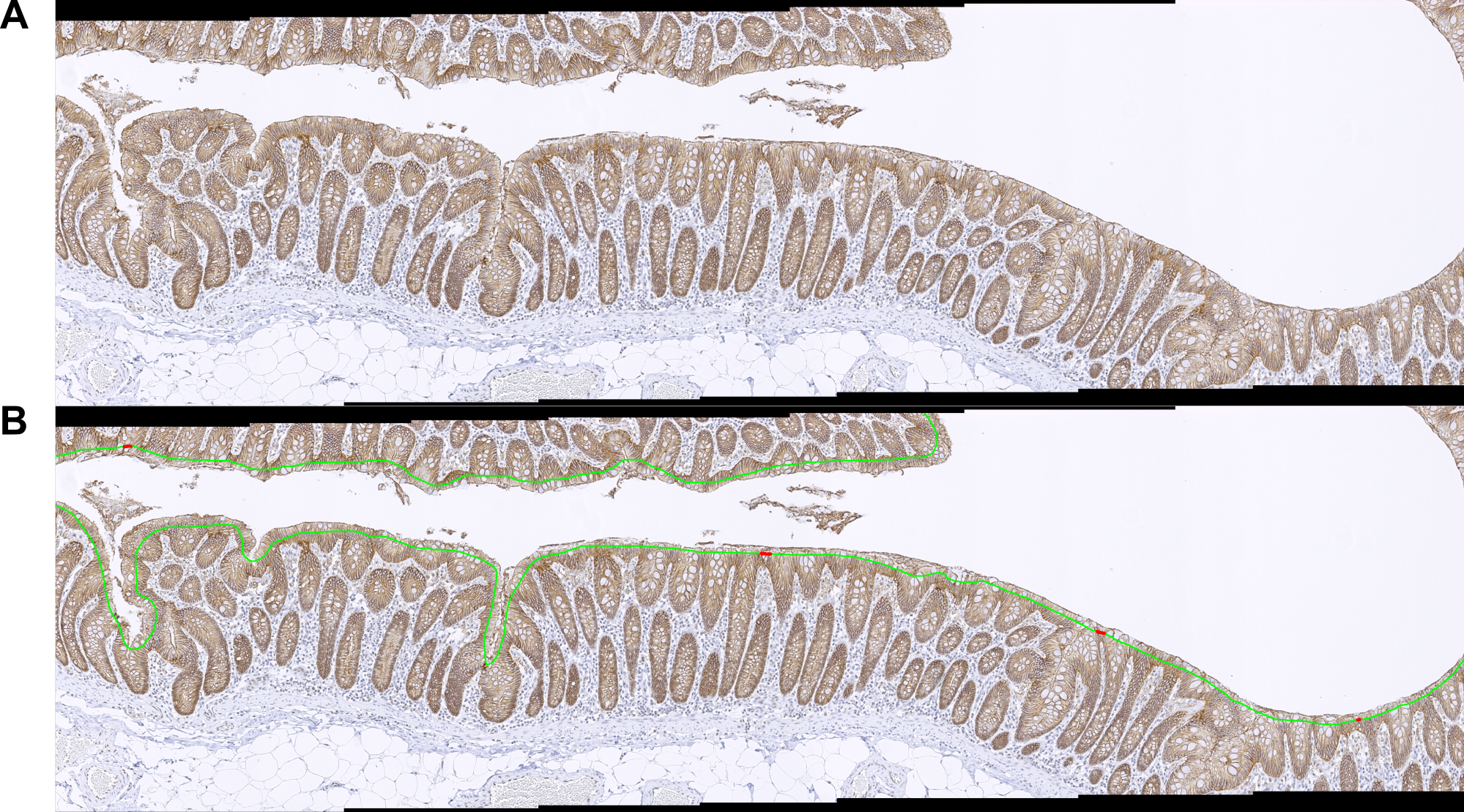

Supplement: S12 Fig — To determine the relative proportion of intact colon mucosal epithelium compared to damaged epithelium, multiple contiguous images of a section of colonic mucosa were obtained. The length of the epithelium shown in the generated composite image was then traced using FIJI v.1.0; here, green lines represents intact, continuous epithelium, while red lines indicates broken, discontinuous epithelium. The lines were drawn freehand at a constant width of 10 pixels. Following tracing, the area of the line segments was measured with FIJI and these areas were used to establish a ratio representing of intact versus broken epithelium. All individual images were captured at 100X magnification using an AxioImager M1 brightfield microscope equipped with an AxioCam MRc5. After collection, the images were stitched together to form a composite using the Stitching plugin for FIJI version 1.0. (TIF) [file ppat.1008333.s017.tif]
