# Supplementary material for: African green monkeys avoid SIV disease progression by preventing intestinal dysfunction and maintaining mucosal barrier integrity
Source: PLoS Pathog. 2020 Mar 2;16(3):e1008333. doi: 10.1371/journal.ppat.1008333 (PMC7077871; doi:10.1371/journal.ppat.1008333)
Supplement: Attachment — Submitted filename: PLOSPathogens_ResponsesToReviewers Final.docx [file ppat.1008333.s018.docx]

Dear Editor,

We would like to thank the reviewers for their positive assessments of our manuscript and for their constructive recommendations. Below, we address all the reviewers’ critiques. We also modified the manuscript accordingly. We are confident that, based on the Reviewers’ recommendations, the manuscript now significantly improved.

Below is the point-by-point rebuttal for the Reviewers’ comments.

Part I - Summary

Reviewer #1: This is an important study that describes mechanisms of natural hosts controlling inflammation that, if translatable to humans, could lead to future therapeutic approaches to alleviate disease progression during the acute and early chronic phase of HIV infection. The experiments were conducted well, with analysis of mucosal integrity and activation at several key points in viral pathogenesis. The IHC data for maintenance of tight junctions and lack of fibrosis is very convincing, especially when shown cross-sectionally with RMs in the chronic phase of infection.

Other differences, however, are less clear, due to the way the data was reported. The manuscript could be improved by addressing points described below.

*Thank you very much for your comments. We revised the manuscript to include all of your suggestions, in the measure in which these changes were feasible. We were limited though by sample availability.*

Reviewer #2: The authors address the important question about the role of microbial translocation in HIV infection. Previous studies have already provided evidence, that natural hosts of SIV do not display a disruption of the intestinal barrier nor microbial translocation. However, the previous studies have mostly focused on the chronic phase of infection. To better understand the mechanisms underlying the preservation of the intestinal barrier, here the early phase of infection, which is known to be crucial for the outcome of an infection, was analyzed. The authors performed an exhaustive study on many time points of early infection and in distinct body and intestinal compartments. The study includes phenotypical, transcriptomic and immunohistochemical approaches for the analysis of the tissues. By analyzing in detail the very early phase of infection, they detected two waves of systemic inflammation, the first one very early (days 3-4 p.i.) and the second one coinciding with the viremia peak in the natural hosts. A transcriptomic profiling provided interesting gene candidates that need to be further studied. The discussion is long but very well written. While this study in many parts remain descriptive, it defines parameters of the very early phases of infection in lymph nodes and gut of a nonpathogenic SIV infection, and attracts attention to the gut damage which, as has been shown by several studies, are not totally restored by anti-retroviral therapy in people infected by HIV and thus remains a major issue.

*Thank you very much for your appreciation.*

Reviewer #3: In this manuscript by senior authors who have extensively published on SIV in African Green monkeys (AGMs), the authors have conducted an exhaustive analysis of very early events post SIV infection to investigate the early changes in gut mucosal integrity and determine whether breaks in mucosal integrity occur early on and then revert to normal or never occur. Serial necropsies were performed on a total of 33 AGMs at time-points 1-3 days post infection, 4-6 dpi, 9-12 dpi and 46-55 dpi to correspond to preramp up, ramp up, peak viremia and early chronic infection. Findings are largely negative. Of major concern is that previously reported findings in natural hosts such as peripheral blood and gut CD4 depletion, acute immune activation are not observed in this study. The authors acknowledge this in the discussion and attribute it to the wide baseline inter-animal variability possibly masking differences in a group comparison. The analysis throughout the manuscript is shown as grouped comparisons of all animals at baseline vs the animals sacrificed at each time-point. Paired comparisons of baseline vs post SIV infection time-point of each cohort is warranted for all the immune parameters analyzed but is not shown.

*Thank you very much for your suggestions, they are addressed below and incorporated in the manuscript whenever possible.*

*We would like to thank the all Reviewers for their kind appreciation of our work and their helpful feedback.*

Other comments:

1. Plasma viral load data should be shown. Figure 1 title states “Blood, jejunum and transverse colon tissue viral loads…..” but shows jejunum, transverse colon and axillary lymph node data.

*The AGM plasma viral loads have been added to Figure 1. More detailed viral loads will be included in future publications and some of the RM viral loads were previously published; please see (PMID: 31704931, Fig 1) for plasma viral loads and (PMID:* 24807713*, Fig 1) for rectal viral loads.*

2. The choice of tissues for viral load estimation is not provided. The reason for selecting axillary lymph node and not showing data on draining lymph nodes or rectal / descending colon tissue after intrarectal inoculation is not evident.

*The reasoning for choosing these tissues was due to the need to match the viral loads with the tissues investigated for mucosal damage. The choice of these tissues was intended to focus this study only on the systematic impact of SIV infection. Axillary LN, jejunum and transverse colon are representative of systemic infection. We reasoned that examination of the site of inoculation or the draining LNs do not reflect as well the effects of systemic SIV infection, especially since the site of inoculation can be subject to perturbations caused by the inoculum. Also, the study of the very early events at the site of inoculation will be the subject of a different manuscript.*

3. It would be helpful if the same y-axes scale is used for all the plots in Figure 1.

*The scale of the y-axes has been modified to match on all the plots. The distribution of the preramp-up points has also been modified to match on all the plots.*

4. Figure 4 shows persistent elevated cytokine levels at the early chronic infection time-point (Figure 4), another unexplained discrepancy from previous reports in this model.

*We included the historical controls, which are late chronic (180 dpi), to demonstrate that even if there is a discrepancy at the set-point, the elevated cytokine levels are normalized during the chronic infection. Also, note that all previous studies of plasma cytokine levels following SIVagm infection in AGMs employed an intravenous route of inoculation with a very large inocula. The intrarectal route of transmission and physiologically relevant dosage of inoculum used in this study may prevent any direct correlation with previous studies.*

5. Rhesus or pig-tailed macaque comparison at similar time-points would have been very useful

*While we fully agree that it would be more informative to have RM or PTM data for similar time points, we do not have precisely matched data from those species for every timepoint. We do not routinely sample such early time points and we did not perform similar early event terminal studies in these species. The terminal nature of this study design also means it is not feasible to obtain new samples. The rationale for not including RM or PTM controls for this study is that there have been multiple publications on the early events of SIV infection in macaques previously. Repeating much of the work that has been done on RMs/PTMs in this study, which is focused on AGMs, would be a major extension in terms of both time and cost with no obvious benefit. We felt such an investment would be better served as a separate, future study rather than an expansion of the current study.*

Figure S4- shows ramp up and peak in macaques but this data is confined to gut transcriptomics. Viral RNA data and immune parameter data etc should also be shown for this group. The methods do not provide any information on the macaque cohort.

*The plasma viral load data, tissue viral load data, methods and information regarding the RMs used for the transcriptomic analysis have been previously published (PMID:* 24807713 *and PMID: 31704931)*

Part II – Major Issues: Key Experiments Required for Acceptance

Reviewer #1

1. Figure 4 depicts fold changes of cytokines from baseline following SIVsab infection. It is unclear what “baseline” refers to here, since what indicated as baseline timepoint in the figure (0 dpi) already has a fold change. Is “baseline” the first access, D -50 pre-infection? If that is the case, how the authors interpret the presence of significant changes in the levels of several cytokines between 0 dpi and the “baseline” timepoint?

*The “Baseline” group here represents animals that were sacrificed uninfected. We have elected to remove the “Baseline” animals from this Figure, since it is principally concerned with changes in response to SIV infection. A new version of the Figure has been added to the paper. In the original submission, the comparison was included between preinfection time points (-50 dpi) and the time of necropsy to illustrate the natural variations in cytokine levels.*

2. Similar comment applies to Figure 9, showing a heatmap of gene expression changes in the gut during SIV infection. It is unclear what they are comparing the pre-ramp, ramp-up, peak, and SP gene expression to, as they are showing baseline gene expression variation as well. Again, it is unclear what “baseline” refers to here.

*The “Baseline” in this Figure refers to the changes in gene expression between each of the individual animals preinfection versus the averages for the entire preinfection group. The changes in the other groups represent the change in gene expression post-infection for each animal compared to the same preinfection group averages. This visualization shows how the genetic responses to infection compares to-individual genetic variation at pre-infection. To avoid confusion, we have changed “Baseline” to “Pre-challenge” in the Figure. We have also added a brief explanation for this to the Legend for Figure 9 (Lines 1620-1625)*

a. The authors should also add the gene expression for MX1 to the main manuscript heatmap, currently MX1 is only shown via IHC and in Fig S4 and gene expression data would strengthen their claim.

*MX1 has been added to Figure 9 as requested, along with a selection of other genes that were associated with “Antiviral Immunity”, to illustrate the degree of MX1 expression. We have also added a new paragraph in the results to address the addition (Lines 383-392).*

b. Based on the RNAseq data in Fig S4, there also seems to be a significant amount MX1 mRNA expression during infection that remains elevated at SP. This is different to what is seen via IHC where at SP, MX1 returns to baseline value. Can the authors comment on this discrepancy?

*At viral set point, MX1 is significantly higher than pre-challenge (p = 0.0082) but not after adjustment for multiple comparisons (adjusted p = 0.082). The log2 fold change is 2.06. By comparison, at D9 and D12, the log2 fold change is 6.96 and 7.34, respectively. The color scale in the figure is balanced to show the numeric range with the most variation (-1.5 to 1.5), which is why high values look similar.* *We have provided a select section of the heatmap to illustrate this point:*

*In summary, we interpret these observations as although some cells expressing the MX1 gene are still present at viral set point, this is not sufficient to cause widespread MX1 tissue expression. We have added a paragraph to the Discussion to address this point (Lines 573-583).*

3. When describing the lack of MT in AGMs it would also be advantageous to show levels of plasma LPS, LBP, sCD14, and EndoCAb which was described in the first MT paper by Brenchley, J. et al. Nat Med 2006. In this study, the authors utilized IHC to look at the core region of LPS and an anti-E. coli antibody to measure enterobacteria species; although that is a very important analyses, plasma levels of the above mentioned soluble markers will contribute to fully address a lack of MT in AGMs.

*While we did not test for levels of LBP and EndoCAb, using ELISA, we found that there were no significant fold changes in the levels of LPS, sCD14, CRP and sP-selectin in infected AGMs versus the baseline timepoints from the same animals, apart from a transient decrease in sCD14. Our group and others have used some or all of these proteins as markers of intestinal dysfunction and microbial translocation in previous studies. Our findings also agree with both data from the IHC for LPS and E. coli, as well as the ELISA for I-FABP, which is more recently described marker of intestinal dysfunction. This data is now shown in Figure S4. We have also added a section to the Results detailing these data (Lines 326-337) and a section to the Methods covering these assays (Lines 855-865).*

Reviewer #2

General statements or extrapolations such as "little to no increase of additional markers of immune activation or inflammation occurred after infection" and "no significant alterations in genes related to immune activation...could be documented" should be removed from the abstract. This does not reduce the interest of the study and only increases its accuracy.

*We have addressed these issues by changing and removing those statements from the abstract.*

Reviewer #3

1. Data re-analyzed to show paired comparisons rather than group analysis.

*In this study design, each of the AGMs was sampled prior to infection, with the sampling consisting of a blood draw, an axillary LN biopsy and a rectal endoscopic biopsy. Because we also needed to provide tissue for RNAseq and the amount of tissue was very small, rectal biopsies were not fixed for IHC. This makes it impossible to compare rectum pre-and postinfection in the same animal. The jejunum and colon were not sampled at all preinfection, so comparisons could also only be done between the uninfected control group and the remaining groups. We did do a pairwise comparison where this was possible. With our techniques, there were enough cells obtained from the preinfection blood and axillary LN biopsy to perform flow cytometry analysis (the rectal biopsy did not yield sufficient cells for staining). This allowed us to use the nonparametric two-tailed Wilcoxon matched-pairs signed ranks test, but only for the blood and axillary LN flow cytometry data.*

2. Comparison with macaques.

*Please see above regarding the challenges around any additional comparisons with RMs.*

Part III – Minor Issues: Editorial and Data Presentation Modifications

Reviewer #1

1. A schematic depicting collections and endpoints would be highly useful, as it avoids having the reader scrolling back to the intro to determine what the timepoints refer to in the subsequent figures.

*We have added a supplemental Figure (Figure S1) depicting the number of animals sampled. Note that each animal was only sampled twice; once prior to infection and then post infection, at the time of necropsy.*

2. Plasma viral loads were not shown in this study. It would be informative to show, particularly considering the variability in tissue viremia among the animals showed in Figure 1. For example, at peak of infection AGM24 was undetectable for both SIV-DNA and SIV-RNA in the transverse colon. Showing plasma viral loads would be helpful to compare infection between all animals.

*The AGM plasma viral loads have been added to Figure 1 and a new paragraph addressing the viral loads has been added to the Results (Lines 216-221). The RM plasma viral loads have been previously published (PMID:* 24807713 *and PMID: 31704931).*

3. Representative flow cytometry plots for Ki-67, CD69, and HLA-DR/CD38 expression should also be shown in the supplemental figures as this is one of the readouts for inflammation after infection.

*We have prepared a new version of Figure S7 with additional flow cytometry plots for Ki-67, CD69 and HLADR/CD38 to demonstrate the strategy.*

*Please note that during the preparation of this new Figure, we observed that the previous gating strategy for HLA-DR/CD38 did not match the current strategy used in our lab. As the original gating was done a long time ago, it is likely that the original strategy was incorrect. So, we redid the analysis for HLA-DR/CD38 for both CD4^+^ and CD8^+^ cells. Both the flow data in Figure 5 and flow cytometry plots in Figure S7 have been updated to reflect this change. We have modified the Results to reflect this (Lines 314-317) and the Legend for Figure S7 (Lines 1785-1791) to accommodate the change.*

4. Materials and methods:

a. Characteristics of the animals (RMs and AGMs) should be included in material and methods (sex, MHC status, age).

*We have compiled as much data as we could find about the animal characteristics. The RM table (Table S1) has been updated and the new table for the AGMs (Table S2) has been added. Please note that records were not complete for all animals or could not be found.*

b. Line 716 is titled as “Tissue VLs quantification”, however, it was described as “Viral RNA was extracted from plasma…”.

*We apologize for this error. We have corrected this and added sections to Methods to detail the plasma viral RNA extraction (Lines 758-763) and the plasma VL quantification (Lines 786-799). The tissue VL extraction is now found on Lines 765-784 and the tissue VL quantification is now found on Lines 801-815.*

c. List flow cytometry mAbs used for the study (fluorophore, clone, and company), similar to what the authors have for IHC antibodies (Table S3).

*We have added a Table (Table S4) listing all the antibodies used for flow.*

5. Figure 9. For the gene network pathway, downregulated genes should be shown in blue to complement the heatmap in Fig. 9A.

*We have altered the colors of the network to reflect the heatmap.*

Reviewer #2: Line 621 : It is stated «that the lack of damage in the gut… are the main factors behind the control of immune activation .. and lack of disease progression in the natural hosts….While microbial translocation might indeed be a major factor in driving chronic inflammation, this study is not designed to compare the impact of the multiple factors to each other and more caution is required.

*We apologize for this oversight. We have modified the final statement (Lines 681-687) to acknowledge the fact that this study only addresses some of the factors that can impact disease progression in natural hosts.*

Lines 137-141 : additional factors have been described, such as the use of CXCR6 that might explain the relative protection of central memory CD4+T cells from SIV infection in natural hosts (Wetzel et al).

*We apologize for this oversight and have added this information to the text along with the relevant citation.*

Line 544 contains a citation of a manuscript that is in revision.

*This manuscript has been now published and the citation has been updated to reflect this.*

Indicate the scales on the images. Indicate how many animals and how many sections per tissue were studied for the image analyses.

*We have added scale bars to all relevant images. We have modified the Methods section to include information about the number of animal and the number of sections and the number of images for quantification analysis.*
